# Responses Toward Injustice Shaped by Justice Sensitivity – Evidence From Germany

**DOI:** 10.3389/fpsyg.2022.858291

**Published:** 2022-08-10

**Authors:** Rebecca Bondü, Anna K. Holl, Denny Trommler, Manfred J. Schmitt

**Affiliations:** ^1^Department of Psychology, Psychologische Hochschule Berlin, Berlin, Germany; ^2^Department of Psychology, University of Potsdam, Potsdam, Germany; ^3^Department of Psychology, University of Konstanz, Konstanz, Germany; ^4^Department of Psychology, University of Koblenz and Landau, Landau, Germany

**Keywords:** justice sensitivity, anger, sadness, helplessness, social withdrawal

## Abstract

Anger, indignation, guilt, rumination, victim compensation, and perpetrator punishment are considered primary responses associated with justice sensitivity (JS). However, injustice and high JS may predispose to further responses. We had *N* = 293 adults rate their JS, 17 potential responses toward 12 unjust scenarios from the victim’s, observer’s, beneficiary’s, and perpetrator’s perspectives, and several control variables. Unjust situations generally elicited many affective, cognitive, and behavioral responses. JS generally predisposed to strong affective responses toward injustice, including sadness, pity, disappointment, and helplessness. It impaired trivialization, victim-blaming, or justification, which may otherwise help cope with injustice. It predisposed to conflict solutions and victim compensation. Particularly victim and beneficiary JS had stronger effects in unjust situations from the corresponding perspective. These findings add to a better understanding of the main and interaction effects of unjust situations from different perspectives and the JS facets, differences between the JS facets, as well as the links between JS and behavior and well-being.

## Introduction

Being sensitive to injustice is considered to predispose to adverse responses toward perceptions of injustice. Rumination is considered the primary cognitive response and perpetrator punishment and/or victim compensation are considered the primary behavioral responses among individuals high in justice sensitivity (JS). The primary affective response depends on the JS facet (victim: anger; observer: indignation; beneficiary/perpetrator: guilt; Schmitt et al., 1995). Research, however, has linked experiences of injustice to further responses (Mikula et al., 1998) that were not yet considered with regard to JS.

We, therefore, examined 17 potential affective, cognitive, and behavioral responses toward unjust situations from all four perspectives (i.e., those of the victim, observer, beneficiary, and perpetrator; Mikula, 1994) that are typical responses toward adverse social experiences in general and toward injustice in particular and that may help to explain maladaptive behavior and impaired well-being among individuals high in JS. In addition, we considered numerous control variables that could also explain the affective, cognitive, or behavioral responses to injustice. Thus, we aimed to add to a more comprehensive understanding of JS and its links with a broader range of emotions, cognitions, and behavior than were previously explored and to gain deeper insights into the differences between the four JS facets. To achieve these aims, reactions to injustice were decomposed into (1) main effects of the perspective from which injustice is perceived (i.e., the characteristics of the situation), (2) main effects of the JS facets (i.e., the characteristics of the person), and (3) interaction effects between characteristics of the situation (i.e., the perspective from which injustice is perceived) and the person (i.e., the four JS facets).

### Justice Sensitivity

Justice sensitivity captures stable individual differences in the tendency to perceive and adversely respond to injustice (Schmitt et al., 1995). Individuals high in JS tend to frequently perceive, ruminate about, and have the urge to level out injustice by victim compensation (particularly individuals high in the observer, beneficiary, and perpetrator JS—the altruistic JS facets) or perpetrator punishment (particularly individuals high in victim JS—the egoistic JS facet; Schmitt et al., 1995). They are hyper-vigilant toward justice-related cues and tend to interpret even ambiguous situations as unjust (Baumert and Schmitt, 2009; Baumert et al., 2012).

The primary affective response depends on the JS facet: highly victim-sensitive individuals (who frequently perceive injustice to their own disadvantage) tend to respond with anger. Highly observer-sensitive individuals (who frequently perceive injustice to the disadvantage of others) with indignation. Highly beneficiary-sensitive individuals (who dislike injustice to their own advantage) and highly perpetrator-sensitive individuals (who fear causing injustice) tend to respond with guilt (Schmitt et al., 1995, 2005, 2010; Schmitt, 1996).

Given similar affective responses, there are strong theoretical and empirical overlaps between beneficiary and perpetrator JS and recent research has sometimes combined observer, beneficiary, and/or perpetrator JS into a single factor of altruistic JS (Fetchenhauer and Huang, 2004; Strauß and Bondü, 2022). All JS facets are positively correlated due to a common underlying concern for justice. The correlations between the three altruistic JS facets are particularly pronounced and ranged between 0.5 and 0.7 (Schmitt et al., 2005, 2010; Baumert et al., 2014; Bondü and Elsner, 2015). Nonetheless, a factor structure of positively related, but separable facets could be established in different age-groups and with different JS measures (Schmitt et al., 2005, 2010; Baumert et al., 2014; Bondü and Elsner, 2015; Strauß et al., 2021).

Victim JS has reliably been related to a broad range of negative outcomes, including aggressive and uncooperative behavior, less sharing, justifications for norm transgressions, and externalizing problem behavior (Fetchenhauer and Huang, 2004; Gollwitzer et al., 2005; Baumert et al., 2014; Bondü and Elsner, 2015; Bondü, 2018). In contrast, the altruistic JS facets have reliably been related to more advantageous outcomes, including prosocial, cooperative, and moral behavior (Fetchenhauer and Huang, 2004; Gollwitzer et al., 2009; Baumert et al., 2014; Strauß and Bondü, 2022). Research has offered different approaches to explaining these outcomes within the existing theoretical framework of JS (Fetchenhauer and Huang, 2004; Gollwitzer et al., 2005, 2013; Strauß and Bondü, 2022).

#### Empirical Findings on Justice Sensitivity That Require Explanation

There are also associations between the JS facets and behavioral as well as mental health outcomes that are not yet well understood, contradicted expectations, and/or that cannot be well explained within the existing theoretical framework of JS and associated approaches. In these cases, a more thorough overview of the affective, cognitive, and behavioral responses associated with the JS facets may help to better understand and explain these associations. For example, first, victim JS was reliably related to anger, revenge, and antisocial behavior (Schmitt, 1996; Fetchenhauer and Huang, 2004; Baumert et al., 2014; Bondü and Richter, 2016). It, however, also showed positive relations with social skills, such as empathy and/or Theory of Mind (Edele et al., 2013; Baumert et al., 2014; Strauß et al., 2021), which are generally thought to protect from antisocial conduct. Results from the present study may, therefore, help to further explain these differential relations. As a second example, although observer and beneficiary JS were generally positively related to prosocial behavior, both were also positively related to perpetrator punishment that may not only be considered an altruistic but also an aggressive act (Lotz et al., 2011; Baumert et al., 2014). In addition, beneficiary JS unexpectedly was positively related to the appreciation of own advantages (that should rather be particularly aversive for individuals high in beneficiary JS) (Pretsch et al., 2016). Furthermore, observer JS was repeatedly positively related to reactive aggression (Bondü and Krahé, 2015; Bondü and Richter, 2016). Finally, research related all JS facets to a broad range of internalizing problems (Bondü et al., 2017, 2020; Bondü and Inerle, 2020; Bilgin et al., 2021), but empirical evidence for the mechanisms potentially explaining these links is sparse.

Therefore, getting deeper insights into typical responses of individuals high in JS may help to explain why they are prone to responding in the observed ways. For example, if individuals high in victim JS not only had a strong tendency to experience anger, but also sadness and disappointment, this finding would help to explain its relations with internalizing symptoms (Bondü et al., 2017; Bilgin et al., 2021); if individuals high in observer JS were found to be prone to helplessness in the present study, this would help to explain its relations with eating disorder pathology (Bondü et al., 2020); if individuals high in beneficiary JS were not as prone to guilt as previously thought, but prone to justifying inequalities, these findings would help to explain why they were found to appreciate rather than dislike own advantages (Pretsch et al., 2016).

#### Differential Relations of the Altruistic Justice Sensitivity Facets

In addition, although the three altruistic JS facets often have similar effects, they were frequently differentially related to outcome measures, including altruistic punishment (Lotz et al., 2011; Baumert et al., 2014), reactive aggression (Bondü and Krahé, 2015; Bondü and Richter, 2016), and moral courage (Niesta Kayser et al., 2010; Baumert et al., 2013). Therefore, it seems important to increase the understanding of how these three JS facets differ in order to explain their diverging effects. For example, it has been assumed that the primary affect associated with both beneficiary and perpetrator JS is guilt. If the present study showed differences in the level of guilt or in the levels of other emotions associated with beneficiary and perpetrator JS, this would help to better carve out the differences between these JS facets. Similarly, victim and observer JS have often shown moderate to high correlations between 0.4 and 0.6 in previous research (Schmitt et al., 2005, 2010; Baumert et al., 2014; Bondü and Elsner, 2015). It has, therefore, been argued that observers may tend to identify with the victims of injustice which would also explain positive relations between observer JS and reactive aggression (Bondü and Krahé, 2015). Similar association patterns of victim and observer JS with the affective, cognitive, and behavioral responses examined in the present study would help to underpin or contradict this notion. In addition, should our analyses indicate influences of victim JS in situations from the observer perspective beyond observer JS, this would also indicate the tendency of observer-sensitive individuals to identify with the victim of the situation.

#### The Role of the Situation

Whereas research on JS systematically captured the disposition to sensitively react to injustice from different perspectives (that is, the JS facets), it did not yet systematically consider the potential role of the perspective from which injustice is presented. Most research presented unjust scenarios from the victim’s or observer’s perspective (Gollwitzer et al., 2005, 2009; Baumert and Schmitt, 2009; Lotz et al., 2013), and some considered the beneficiary’s and perpetrator’s perspectives (Schmitt, 1998; Maes and Schmitt, 1999; Schmitt et al., 2000; Gollwitzer et al., 2005). According to trait activation theory (Tett and Burnett, 2003), however, the different JS facets should be specifically activated by, and particularly impactful in, situations that correspond with these traits. Correspondence means that a situation contains cues of (in)justice *and* is presented from the perspective that matches one of the JS facets (but not the other three). Consequently, the JS facets should be specifically related to affective, cognitive, and behavioral responses in unjust situations that are perceived from the corresponding perspective. Put differently, interpersonal differences should become particularly relevant in situations that correspond with the given trait. The present study, therefore, systematically crossed situational perspectives and JS facets to close this gap in research.

### Further Potential Responses to Injustice

Previous research has provided abundant evidence on the relations of JS with the aforementioned affective, cognitive, and behavioral responses. However, there may be further relevant responses that were not yet considered with regard to JS, but that were observed in research on responses to injustice, stress, negative mental states in general, or rejection.

#### Affective Responses

Previous research linked experiences of injustice to an array of negative emotions (Mikula et al., 1998; Krehbiel and Cropanzano, 2000). *Anger* is considered the primary affective response to injustice by victims that may warn the perpetrator of future unjust behavior (Averill, 1983; Schmitt and Mohiyeddini, 1996). But anger may not be limited to victims: Observers of injustice are thought to be prone to *indignation*, an emotion close to anger; also, beneficiaries and perpetrators may experience anger/indignation at the situation and/or the perpetrator/themselves.

Particularly victims of injustice can also experience *sadness* and/or *self-pity* when a deserved or desired outcome was not received or when feeling negatively treated by others (Mikula et al., 1998; Stöber, 2003). If others observe, benefit from, or cause injustice and witness the victim’s negative responses, they should be prone to empathize with the victim and, thus, also feel sad and pity the victim. Although particularly beneficiaries and perpetrators should be prone to *guilt*, observers may also feel guilty if they cannot help the victim or when they themselves are better off. Even victims may feel guilty if they blame themselves for the situation (Mikula et al., 1998). Because individuals are generally motivated to avoid injustice, they may experience *helplessness* if they fail to do so. Observers may feel helpless if they cannot prevent injustice or help the victim. If it is difficult or impossible to restore justice, beneficiaries and perpetrators of injustice should feel helpless as well. Finally, all parties should be prone to *disappointment*, for example when promises were broken, positive expectations were not met, or if the perpetrator behaved adversely in another, unexpected way (Schmitt et al., 1995; De Cremer, 2006).

Individuals may be prone to experiencing numerous and diverse negative emotions, for example, if they are high in neuroticism. They may also be prone to experience certain emotions in particular: some individuals may tend to respond with anger, whereas others may be particularly prone to guilt (e.g., Cohen et al., 2012; Miller et al., 2019). The situation may influence the tendency to show specific affective responses as well, for example by promoting different emotions depending on the perspective from which the situation is perceived. Finally, the same situation may trigger different affective responses and different individuals may be more or less prone to some of these responses. For example, the construct of rejection sensitivity distinguishes two facets of angry and anxious rejection sensitivity that may be differentially pronounced in children and adolescents (Downey et al., 1998) and that differentially relate to social behavior and mental health problems (Marston et al., 2010). It is, therefore, important to further disentangle potential personal and situational effects on affective responses associated with JS and unjust situations.

#### Cognitive Responses

Cognitive strategies are powerful means to alter the perception of situations in order to minimize their negative effects and to regulate negative affect in the face of injustice. Cognitive coping or emotion-regulation strategies, however, were hardly examined with regard to JS. In line with general strategies of cognitive emotion regulation (Garnefski et al., 2001) or moral disengagement (Bandura et al., 1996) and irrespective of the perspective from which injustice is perceived, witnesses of injustice may, therefore, revert to *justify* the perpetrator’s behavior, *blame the victim* or *minimize the resulting harm*, in order to relieve the strain caused by perceptions of injustice (Lerner, 1980). Adverse experiences, such as injustice, may also result in *rumination* as a maladaptive emotion-regulation strategy (Olatunji et al., 2013, for a meta-analysis) and difficulties to *suppress* thoughts of the situation. Finally, experiences of injustice may result in the *anticipation of future injustice* (Fetchenhauer and Huang, 2004) that can be associated with worry and fear of own or repeated victimization.

The relations between these responses were hardly examined in previous research (Yang et al., 2021, for a study on the relationship between anger rumination and moral disengagement). For example, it is, therefore, yet unknown, whether individuals who tend to ruminate about injustice should be hampered to reduce the strain associated with experiencing unjust events by employing strategies of moral disengagement or whether these individuals should be particularly in need of and, therefore, prone to use these strategies. Similarly, it has been argued that the anticipation of future injustice may explain adverse behavior among individuals high in victim JS in terms of dysfunctional thoughts (Fetchenhauer and Huang, 2004; Bondü and Elsner, 2015), but this assumption has not been tested. Therefore, more thoroughly examining diverse potential cognitive responses toward injustice may add to a better understanding of the effects of JS.

#### Behavioral Responses

Injustice often urges to restore justice. *Perpetrator punishment* is considered the primary behavioral response particularly of victims of injustice, but also by observers or (involuntary) profiteers. Even perpetrators may revert to self-punishment if victim compensation is impossible (Nelissen and Zeelenberg, 2009). Although particularly observers, beneficiaries, or perpetrators of injustice should strive to *compensate* the victim, victims may also try to do so themselves. Further possibilities to restore justice that were not yet considered with regard to JS include trying to *resolve the conflict* (e.g., by mediating between conflicting parties, excusing, or promising change) or to *forgive* the perpetrator (Deutsch and Coleman, 2000). Finally, individuals may revert to *social withdrawal* in order to prevent themselves from becoming a victim of, witnessing, profiting from, or causing injustice.

Although primary behavioral responses have been attributed to each of the four JS facets, it is not well known how they relate to the other potential responses. For example, individuals high in victim JS should be prone to perpetrator punishment and less prone to forgive (Gerlach et al., 2012). It is, however, not well known whether they should nonetheless be inclined to resolve the conflict at the same time, as may be suggested by higher levels of social skills even among children high in victim JS (Strauß et al., 2021). It is, therefore, important, to more thoroughly investigate these relation patterns to better understand the JS perspectives as well as potential differences between them and how they may affect behavior in different ways.

### Situational Perspective Effects

Many of the aforementioned responses were addressed by previous justice research. However, no study has investigated them simultaneously. More importantly, no previous study systematically investigated how these responses differ in intensity between the perspectives from which injustice can be experienced. Previous research suggests that some responses (e.g., rumination), are general, perspective-independent reactions to injustice, whereas others (e.g., feelings of guilt), more strongly depend on the role a person plays in the incident. Together with a large number of potential responses to injustice, the fully crossed person × situation design of our study allows us to more systematically investigate how the situational perspective affects these responses than was previously done.

### Justice Sensitivity Effects

Given that individuals high in JS value justice particularly highly, adverse responses to injustice should be particularly pronounced among these individuals. In addition to the assumed general associations between JS and adverse responses to injustice, the JS facets should predispose to specific responses. For example, although justification, victim-blaming, or minimization of harm are general responses to injustice (Lerner, 1980), individuals high in altruistic JS may be less able to revert to these coping strategies. Although forgiveness is a common strategy for resolving unjust situations, victim JS was negatively related to dispositional forgiveness (Gerlach et al., 2012).

### Person × Situation Interaction Effects

Trait activation theory predicts that a specific JS facet (e.g., victim JS) will be particularly strongly related to affective, cognitive, and behavioral responses to unjust situations that are perceived from the corresponding perspective (i.e., the victim perspective). By contrast, a JS facet (e.g., victim JS) will be related less strongly to these responses in unjust situations that are experienced from discordant perspectives (here: observer, beneficiary, and perpetrator).

### The Present Research

#### Research Goals

The present research was guided by three main goals. First, we wanted to explore the effects of the situational perspective on a large number of potential affective, cognitive, and behavioral responses to injustice. We wanted to reveal which of these responses are general responses to injustice and which responses are more perspective specific, that is, vary in intensity depending on the perspective from which injustice is experienced (i.e., the main effects of the situational perspective).

Second, we wanted to explore more systematically than before how victim, observer, beneficiary, and perpetrator JS predispose to cognitive, affective, and behavioral responses to injustice, in particular those that have not been linked with JS in previous research (i.e., the main effects of the four JS facets).

**FIGURE 1:**
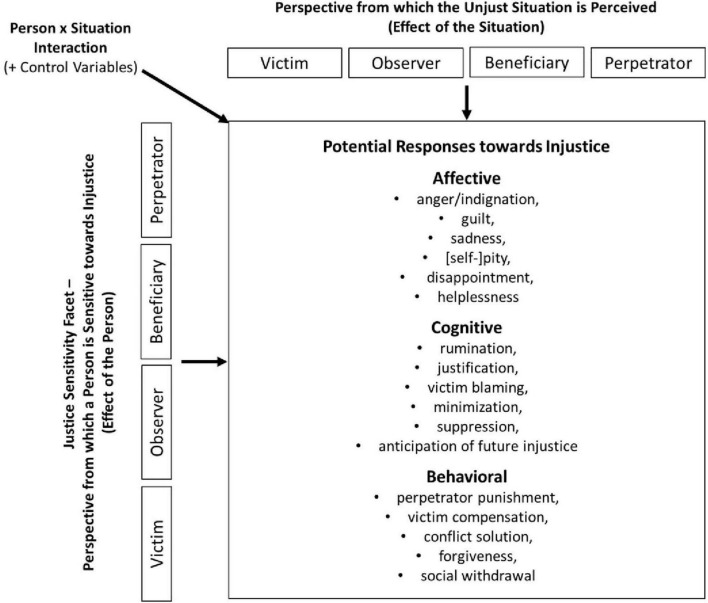
Research design of the current study.

Third, inspired by trait activation theory, we intended to reveal how situational characteristics and characteristics of the person jointly affect responses to injustice. Trait activation theory predicts that JS will impact responses to injustice most forcefully if the perspective from which injustice is experienced (situation) matches the JS facet (person) (i.e., the interaction effect of situation × person; Figure 1).

#### Overview of Study

We recruited a sample of German adults who rated their agreement to 17 potential affective, cognitive, and behavioral responses toward three unjust situations from the victim’s, observer’s, beneficiary’s, and perpetrator’s perspectives, respectively. In doing so, we collected data that allowed us to examine (1) which are the most relevant affective, cognitive, and behavioral responses toward unjust situations in general and from the victim’s, observer’s, beneficiary’s, and perpetrator’s perspectives (situation main effects), (2) how these responses are associated with the JS facets regardless of the situation (personality main effects), (3) and whether the effects of the JS facets are stronger in unjust situations from the corresponding perspective than in unjust situations from perspectives that do not correspond with the JS facet at issue (person × situation interaction effects).

In order to estimate the unique contribution of JS to emotional, cognitive, and behavioral reactions to unjust situations, we controlled for numerous other variables that may also account for differences in these responses and/or that show conceptual overlaps with JS. By way of example, we controlled for trait anger, spite, narcissism, and a tendency toward vengeance, because they may also explain why individuals should be prone to experiencing anger or to seek perpetrator punishment; we controlled for extraversion, self-esteem, and locus of control, because they may influence whether individuals should be prone to socially withdraw or actively engage in social problem solving; we controlled for empathy, because it may explain why individuals high in the altruistic JS facets should be prone to prosocial responses toward the victims of injustice; we controlled for the general tendency toward moral disengagement, because this may also explain the engagement in similar cognitive strategies specifically in unjust situations.

#### Hypotheses

We expected (1) unjust situations to be associated with a broad range of adverse affective, cognitive, and behavioral responses (irrespective of the individual expression of JS), (2) victim, observer, beneficiary, and perpetrator JS to be related to these affective, cognitive, and behavioral responses to injustice (irrespective of the perspective from which the unjust situations are perceived), and (3) the relations of JS with these responses to be more pronounced in situations that were presented from the perspective that corresponds with the respective JS facet (Table 1 shows detailed Hypotheses based on previous research findings and theoretical considerations for the expected situation × person interaction effects; given that unjust situations from a specific perspective and the sensitivity toward injustice from the corresponding JS facet should elicit similar responses as their interaction, we assumed that the situation and person main effects should be equivalent to their interaction effects and only present a single table for the hypotheses). We also explored whether the potential interaction effects held stable when the control variables and the respective other three JS facets were entered into the analyses.

**TABLE 1:** Expected direction of effects of JS facets on responses to unjust situations from the corresponding perspective and indication of supported hypotheses.

	Victim JS	Observer JS	Beneficiary JS	Perpetrator JS
*Anger/Indignation*	+ (✓)	+ ✓	+ ✓	+ ✓
*Guilt*	–	?	+ ✓	+ ✓
Sadness	+ ✓	+ ✓	+ ✓	+ ✓
(Self-)Pity	+ ✓	+ ✓	+ ✓	+ ✓
Disappointment	+ ✓	+ ✓	+ ✓	+ ✓
Helplessness	+ ✓	+ ✓	+ ✓	+
*Rumination*	+ ✓	+ ✓	+ ✓	+ ✓
Justification	–	?	?	?
Victim blaming	–	?	?	?
Trivialization	– ✓	?	?	?
Suppression	–	– ✓	–	–
Anticipation of future injustice	+ ✓	+ ✓	+	+
*Perpetrator punishment*	+ ✓	+	+	+
*Victim compensation*	+ ✓	+ ✓	+ ✓	+ ✓
Conflict solution	–	+ ✓	+ ✓	+ ✓
Forgiveness	– ✓	–	–	–
Social withdrawal	+ ✓	?	?	?

*+ positive relations expected; − negative relations expected; ? no hypothesis; ✓ hypothesis supported; assumed primary responses in italics.*

## Materials and Methods

### Sample

A convenience sample of *N* = 293 participants (83.6% women) between 17 and 70 years of age (*M* = 23.63 years, *SD* = 7.30) took part in the study. Out of these, 96.2% had German citizenship. Students were largely overrepresented (89%); 8% of the participants held a university degree, 88% a university entrance qualification, and 4% a university of applied sciences entrance qualification, vocational level qualification, or another type of graduation.

### Procedure

Participants answered an online survey in 2013 and 2014. They were recruited *via* ads in the university building, social networks, and personal communication. They were informed about the study’s purpose. All attended voluntarily and were guaranteed anonymity. Participants qualified for taking part in a lottery for 10 vouchers from an online retail company. Students at the university could receive course credit. The procedures were in accordance with the 1964 Helsinki declaration and comparable ethical standards. There was no missing data except for three missing data points in the spite items. Due to the very low number of missing data in this control variable, we computed the spite mean value from the remaining five items for these three participants. We did not pre-register our hypotheses, because at the time of the data collection and the first analyses, this was not yet a common proceeding. The data, codebook, and analysis code can be accessed here: https://osf.io/ht97r/?view_only=f92d0886340544da83bffeb205f3cd2e.

### Measures

#### Justice Sensitivity

We measured each JS facet *via* five items from the short version of the Justice Sensitivity Inventory (Schmitt et al., 2005; Bondü and Elsner, 2015; e.g., victim JS: “It makes me angry when I am treated worse than others”; perpetrator JS: “I feel guilty when I treat someone worse than others.”). Response options ranged from 0 (*not applicable at all*) to 5 (*perfectly applicable*). The original and the short scale have been shown to be reliable and valid (Schmitt et al., 2005, 2010; Bondü and Elsner, 2015).

#### Responses to Injustice

Participants were presented with three unjust scenarios per perspective (i.e., the victim, observer, beneficiary, and perpetrator perspective; Appendix A for vignette examples), that is, with 12 scenarios in total. Scenario descriptions were adapted to the sex of the participant. We had pre-tested and pre-selected these scenarios in two pre-tests: First, we collected and thought of descriptions of potentially unjust scenarios and decided from which of the four perspectives these scenarios seemed most realistic. We then presented *N* = 20 individuals between 21 and 75 years of age (*M* = 32.05, *SD* = 13.39; 50% women) with 28 scenarios (seven scenarios from the victim and perpetrator perspectives, respectively; eight scenarios from the observer perspective; six scenarios from the beneficiary perspective) and asked them to rate how unjust, realistic, upsetting, and interesting they found each scenario on 5-point scales, respectively (0—*not at all* to 4—*very*). In a second pre-test with *N* = 18 individuals between 23 and 52 years (*M* = 29.78, *SD* = 8.84; 50% women) we pre-tested nine additional scenarios (one from the victim perspective and four from the beneficiary and perpetrator perspectives, respectively). Out of the 37 scenarios, two of the present authors selected the three scenarios per perspective that were rated as most unjust (main decision criterion) and were at the same time considered realistic.

After each of the 12 scenarios, participants rated how much they agreed that they would show six affective (anger/indignation, guilt, sadness, disappointment, [self-]pity, helplessness), six cognitive (rumination, justification, victim-blaming, trivialization, suppression, anticipation of future injustice), and five behavioral responses (perpetrator punishment, victim compensation, conflict solution, forgiveness, social withdrawal; Appendix B for item wordings) in this situation on a six-point Likert-scale from 0—*not agree at all* to 5—*totally agree*. We computed mean scores for each response across scenarios separately for each perspective.

#### Control Variables

We assessed various control variables to examine whether JS would predict the responses toward injustice beyond these variables. We computed mean scores for all variables.

***Trait anger*** was assessed by eight items of the corresponding subscale of the German State-Trait Anger Expression Inventory-2 (STAXI; Rohrmann et al., 2013; “I get angry easily”; response options: 1—*almost never* to 4—*almost always*).

***Narcissism*** was assessed by five items (“I wish someone would write my biography one day”; response options: 1—*does not apply at all* to 5—*completely applies*) of a German short version of the Narcissistic Personality Inventory (NPI-d5; Collani, 2014).

***Moral disengagement*** was assessed *via* eight items (“Repulsive persons do not have the right to be treated as human beings”; response options: 1—*not agree* to 5—*totally agree*) of the Mechanisms of Moral Disengagement Questionnaire (Bandura et al., 1996).

***Motivation for vengeance*** was assessed *via* eight items (“Vengeance is sweet”; response options: 1—*not agree* to 6—*totally agree*) of the Vengeance Scale (Stuckless and Goranson, 1992).

***Spite*** was assessed *via* six items (“I often reject things on principle”; Case and Fitness, personal information; response options: 1—*not at all agree* to 7—*totally agree*).

***External/internal locus of control*** was assessed *via* four items each (external: “Much of what happens in my life depends on chance”; internal: “Often I do not know how to fulfill my wishes”; Richter et al., 2013; 1—*very wrong* to 6—*very right*).

***Self-esteem*** was assessed *via* 10 items (“I am content with me”) from the Frankfurt Self-Concept Scales (Deusinger, 1986; response options: 1—*not agree* to 6—*fully agree*).

***Empathy*** was assessed *via* eight items (“Before I criticize someone, I try to imagine how it looks from his point of view”; response options: 1—*almost never* to 5—*almost always*) from the Saarbruecker Personality Questionnaire on Empathy (Paulus, 2011).

***Extraversion*** was assessed *via* three items (“I am someone who is communicative, talkative”; 1—*not agree* to 7—*totally agree*) of the German Socio-Economic Panel (Richter et al., 2013).

### Analysis

We tested our hypotheses in three steps: First, we examined the main effect of the situation by comparing the mean values for the 17 affective, cognitive, and behavioral responses (a) averaged across all 12 unjust situations and (b) averaged across the three unjust situations per perspective from which injustice may be perceived. Thus, we examined which responses unjust situations tend to trigger in general and from the four different perspectives in particular, irrespective of whether a person is high or low in JS (situation main effect). We considered the findings to indicate relevant responses to unjust experiences if the average expression was ≥2, that is, above the mean of the scales with a range of 0 to 5.

Second, we examined individual differences in reactions to injustice by inspecting the correlations of the 17 responses to unjust situations with the four JS facets, averaged across all 12 unjust situations. Thus, we examined which responses are related to the different JS facets irrespective of the perspective from which an unjust situation is experienced (personality main effect). We considered the findings to indicate relevant relations if the correlations were significant.

Finally, we estimated person × situation interaction effects *via* path models that predicted the 17 responses to unjust situations from the victim’s, observer’s, beneficiary’s, and perpetrator’s perspectives specifically for the accordant JS facet. In a second step, all discordant JS facets and control variables were entered into these path models in order to estimate the unique effects of the accordant JS facet. Thus, we examined the specific effects of the JS facets in unjust situations from the corresponding perspective and whether they would hold stable beyond the control variables. We considered the findings to indicate relevant relations if the path coefficients were significant.

## Results

### Descriptive Statistics and Correlations

Table 2 shows the internal consistencies, means, and standard deviations of the JS facets and all control variables for the total group and separated by gender. A MANCOVA controlled for age revealed a multivariate effect of gender *F*(14, 277) = 3.45, *p* < 0.001, $\eta_{p}^{2}$ = 0.148. Subsequent ANCOVAs showed higher observer (*p* = 0.02), beneficiary (*p* < 0.001), and perpetrator JS (*p* < 0.001), higher empathy (*p* = 0.004) and lower motivation for vengeance (*p* < 0.001), moral disengagement (*p* = 0.029), as well as narcissism (*p* < 0.001) in women than in men. Men were significantly older than women (*t* = 3.525, *df* = 291, *p* < 0.001, $\eta_{p}^{2}$ = 0.041).

**TABLE 2:** Descriptive statistics for JS facets and control variables and correlations between JS facets and control variables.

	Cronbach’s α	Total *M* (*SD*)	Women *M* (*SD*)	Men *M* (*SD*)	Victim JS	Observer JS	Beneficiary JS	Perpetrator JS
Victim JS	0.78	3.42 (0.86)	3.46 (0.84)	3.24 (0.93)		0.33***	0.08	0.10
Observer JS*	0.86	3.43 (0.90)	3.48 (0.88)	3.16 (0.98)			0.58***	0.57***
Beneficiary JS**	0.85	3.24 (0.96)	3.32 (0.96)	2.86 (0.91)				0.74***
Perpetrator JS***	0.86	3.74 (0.94)	3.85 (0.88)	3.18 (1.03)				
Trait anger	0.81	1.94 (0.52)	1.94 (0.51)	1.93 (0.56)	0.32**	0.07	−0.12*	−0.19**
Narcissism**	0.80	2.81 (0.85)	2.75 (0.85)	3.16 (0.75)	0.22**	0.04	−0.13*	−0.11
Moral disengagement	0.54	2.23 (0.50)	2.20 (0.49)	2.34 (0.54)	0.06	−0.13*	−0.24***	−0.27***
Vengeance***	0.78	2.57 (0.77)	2.49 (0.69)	2.98 (1.02)	0.12*	−0.14*	−0.23***	−0.37***
Spite	0.73	3.27 (1.05)	3.24 (1.04)	3.42 (1.11)	0.16**	−0.06	−0.16**	−0.25***
External locus of control	0.50	3.47 (0.73)	3.50 (0.73)	3.34 (0.71)	0.25**	0.09	0.08	0.02
Internal locus of control	0.56	3.92 (0.69)	3.90 (0.70)	4.06 (0.67)	−0.10	−0.05	−0.04	0.00
Self-esteem	0.90	4.74 (0.82)	4.71 (0.84)	4.92 (0.70)	−0.07	−0.04	−0.09	0.01
Empathy**	0.71	3.84 (0.46)	3.87 (0.44)	3.66 (0.49)	0.11	0.38***	0.37***	0.45***
Extraversion	0.84	4.80 (1.29)	4.82 (1.27)	4.67 (1.41)	0.01	0.08	−0.02	0.07
Age***	–	23.63 (7.30)	22.98 (6.85)	26.96 (8.60)	−0.20***	0.01	0.14*	0.01

*Significant differences between women and men in a MANCOVA, F(14, 277) = 3.45, p < 0.001.*

***p < 0.001, **p < 0.01, *p < 0.05.

Observer, beneficiary, and perpetrator JS were closely correlated (*r* = 0.57–0.74; Table 2). Victim JS showed positive correlations with observer JS, trait anger, narcissism, motivation for vengeance, spite, and external locus of control. Beneficiary and perpetrator JS showed negative correlations with trait anger and narcissism. Observer, beneficiary, and perpetrator JS showed negative correlations with moral disengagement, vengeance, and spite, and positive correlations with empathy. Victim JS was negatively related to age, whereas beneficiary JS was positively related to age.

### Situation (Perspective) Main Effects on Responses to Injustice

Averaged across *all 12 unjust situations* from all four perspectives, all six affective responses in the current study showed average expressions ≥ 2 with the highest expressions for anger, pity, and disappointment. Concerning the cognitive responses, rumination and anticipation of future injustice showed average expressions ≥ 2, but not justification, victim-blaming, trivialization, and suppression. Concerning behavioral responses, conflict solution, victim compensation, and forgiveness showed average expressions ≥ 2, but not perpetrator punishment and social withdrawal.

*For situations from the victim’s perspective*, all affective responses but guilt showed above-average expressions ≥ 2. Anger and disappointment stood out as the most relevant affective responses, followed by sadness. Concerning cognitive responses, rumination and anticipation of future injustice showed average expressions ≥ 2. Concerning behavioral responses, conflict solutions showed average expressions ≥ 2.

*For situations from the observer’s perspective*, all affective responses but guilt showed average expressions ≥ 2. Anger and pity stood out as the most relevant affective responses, followed by disappointment. Concerning cognitive responses, rumination and anticipation of future injustice showed average expressions ≥ 2 with an emphasis on rumination. Concerning behavioral responses, conflict solution and victim compensation showed average expressions ≥ 2.

*For situations from the beneficiary’s perspective*, all affective responses but sadness showed average expressions ≥ 2. Pity and anger were the most relevant. Concerning cognitive responses, rumination, suppression, and anticipation of future injustice showed average expressions ≥ 2, with a slight emphasis on anticipation of future injustice. Concerning behavioral responses, conflict solution and forgiveness showed average expressions ≥ 2.

*For situations from the perpetrator’s perspective*, all affective responses showed average expressions ≥ 2. Guilt, pity, and disappointment were most relevant. Concerning cognitive responses, rumination, and anticipation of future injustice showed average expressions ≥ 2, with an emphasis on rumination. Concerning behavioral responses, victim compensation, conflict solution, and forgiveness showed average expressions ≥ 2 with an emphasis on conflict solution (Table 3 for all results).

**TABLE 3:** Mean responses to injustice averaged across all 12 situations (total) and averaged across the three situations per justice-sensitivity perspective.

	Total *M (SD)*	Victim *M (SD)*	Observer *M (SD)*	Beneficiary *M (SD)*	Perpetrator *M (SD)*
*Anger/Indignation*	3.77 (0.62)	4.48 (0.68)	4.33 (0.71)	3.44 (0.96)	2.84 (1.30)
*Guilt*	2.05 (0.62)	0.68 (0.80)	0.73 (0.86)	2.47 (1.30)	4.34 (0.84)
Sadness	2.61 (1.05)	3.07 (1.29)	2.93 (1.25)	1.88 (1.24)	2.55 (1.32)
(Self-)Pity	3.74 (0.76)	2.73 (1.38)	4.33 (0.72)	3.81 (0.91)	4.09 (0.89)
Disappointment	3.65 (0.79)	4.16 (0.84)	3.40 (1.09)	2.98 (1.21)	4.07 (0.93)
Helplessness	2.69 (0.96)	2.81 (1.15)	2.95 (1.16)	2.98 (1.18)	2.03 (1.31)
*Rumination*	3.50 (0.82)	3.89 (0.93)	3.26 (0.95)	2.87 (1.12)	3.99 (0.94)
Justification	1.22 (0.63)	0.89 (0.77)	0.82 (0.73)	1.53 (0.94)	1.63 (0.98)
Victim blaming	0.74 (0.53)	1.18 (0.99)	0.38 (0.52)	0.69 (0.70)	0.71 (0.69)
Trivialization	1.22 (0.61)	1.15 (0.92)	1.02 (0.77)	1.89 (0.99)	0.79 (0.74)
Suppression	1.49 (0.84)	1.36 (1.02)	1.40 (0.93)	2.04 (1.21)	1.17 (1.07)
Anticipation of future injustice	2.82 (1.01)	3.64 (1.13)	2.17 (1.08)	3.17 (1.19)	2.31 (1.40)
*Perpetrator punishment*	1.17 (0.79)	1.74 (1.25)	0.89 (1.03)	0.62 (0.73)	1.43 (1.28)
*Victim compensation*	2.41 (0.84)	1.89 (1.40)	2.05 (1.14)	1.73 (1.17)	3.96 (0.83)
Conflict solution	3.34 (0.73)	3.64 (0.97)	2.78 (1.13)	2.60 (1.20)	4.35 (0.77)
Forgiveness	2.25 (0.83)	1.86 (1.06)	1.65 (1.03)	2.62 (1.00)	2.85 (1.09)
Social withdrawal	1.08 (0.87)	1.67 (1.26)	1.22 (1.03)	0.83 (0.83)	0.60 (0.81)

*Range of all variables: 0 to 5; assumed primary responses in italics.*

### Personality (Justice Sensitivity) Main Effects on Responses to Injustice

Personality main effects are reported in Table 4 as zero-order correlations between the JS facets and the 17 responses to injustice averaged across all four perspectives from which injustice can be perceived (i.e., averageed across all 12 unjust situations in our study). All JS facets were positively related to all negative emotions, rumination, and victim compensation. In addition, victim JS was positively related to anticipation of future injustice, perpetrator punishment, and social withdrawal; observer JS was positively related to anticipation of future injustice and conflict solution and negatively related to suppression; beneficiary JS was positively related to conflict solution and negatively related to trivialization and suppression; perpetrator JS was positively related to conflict solution and negatively related to victim-blaming, trivialization, suppression, and perpetrator punishment. Thus, complementing previous JS research, JS showed positive links with numerous negative responses—including responses not yet considered in previous research—to unjust situations regardless of the perspective from which they were perceived.

**TABLE 4:** Zero-order correlations between JS facets and responses to injustice averaged across all 12 unjust situations.

	Victim JS	Observer JS	Beneficiary JS	Perpetrator JS
*Anger/Indignation*	0.291***	0.320***	0.215***	0.310***
*Guilt*	0.159**	0.292***	0.330***	0.356***
Sadness	0.232***	0.360***	0.311***	0.350***
(Self-)Pity	0.338***	0.271***	0.131*	0.252***
Disappointment	0.316***	0.303***	0.275***	0.302***
Helplessness	0.282***	0.188***	0.159**	0.233***
*Rumination*	0.213***	0.298***	0.343***	0.358***
Justification	–	–	–	–
Victim blaming	–	–	–	−0.129*
Trivialization	–	–	−0.163**	−0.128*
Suppression	–	−0.120*	−0.217***	−0.165**
Anticipation of future injustice	0.334***	0.219***	–	0.116*
*Perpetrator punishment*	0.213***	–	–	−0.171**
*Victim compensation*	0.166**	0.276***	0.218***	0.192***
Conflict solution	–	0.203***	0.197***	0.210***
Forgiveness	–	–	–	–
Social withdrawal	0.194***	–	–	–

Only significant correlations displayed; ***p < 0.001, **p < 0.01, *p < 0.05 assumed primary responses in italics.

When comparing the strengths of associations, victim JS was the facet most closely related to self-pity, disappointment, helplessness, the anticipation of future injustice, perpetrator punishment, and social withdrawal (all positive); observer JS was most closely related to indignation, sadness, and victim compensation (all positive); beneficiary JS was most closely (negatively) related to trivialization and suppression; perpetrator JS was most closely related to guilt, rumination, conflict solution (all positive), and victim-blaming (negative).

### Person (Justice Sensitivity) × Situation (Perspective) Interaction Effects on Responses to Injustice

Tables 5–8 show the results of the prediction of all responses to unjust situations when described from the victim, observer, beneficiary, and perpetrator perspectives. The figures before the slashes in these tables represent the standardized simple regression weights of the JS facet at issue without controlling for the other JS facets and the control variables. The figures behind the slashes represent the standardized multiple regression weights of the JS facet at issue when controlling for all other variables including gender and age (see Supplementary material for all significant path coefficients including control variables). The second multiple regression weight thus reflects the unique contribution of the JS facet at issue to the prediction of the response at issue in unjust situations described from the corresponding perspective. Due to limited space, we cannot describe and comment on each effect reported in Tables 5–8. Rather, we will provide a summary, first, of the overall effect pattern and, second, separately for each JS facet.

**TABLE 5:** Prediction of responses in situations from the victim’s perspective by the JS facets when including only victim JS/all variables.

	Victim situation × victim JS	Victim situation × observer JS	Victim situation × beneficiary JS	Victim situation × perpetrator JS
*Anger/Indignation*	0.460***/0.324*		–/−0.226**	–/0.194*
*Guilt*	–/–		–/0.199**	
Sadness	0.265***/0.175**			–/0.238*
(Self-)Pity	0.388***/0.262***			
Disappointment	0.356***/0.282***			
Helplessness	0.254***/0.156*			
*Rumination*	0.239***/0.182**			
Justification	–/−0.189**			
Victim blaming	–/–			
Trivialization	−0.223***/−0.231***			
Suppression	–/–			
Anticipation of future injustice	0.374***/0.260***			
*Perpetrator punishment*	0.329***/0.213***			
*Victim compensation*	0.216***/–			
Conflict solution	–/–			–/0.218*
Forgiveness	−0.218***/−0.238***	–/0.187**		
Social withdrawal	0.232***/0.127*			

Only significant path coefficients displayed; ***p < 0.001, **p < 0.01, *p < 0.05 assumed primary responses in italics.

Overall and in line with trait activation theory and the person × situation interaction effects implied by this theory, regression effects of the four JS facets on the 17 responses to injustice tended to be stronger in scenarios that described injustice from the perspective that matched the JS facet than in scenarios from the other perspectives. However, the relations with responses in scenarios from the perspective that matched the JS facet were only stronger than the relations to responses across all unjust scenarios for the victim and beneficiary JS and tended to be lower for the observer and perpetrator JS. This indicates that only victim and beneficiary JS have specific effects in unjust situations that match the perspective of these traits.

Concerning JS-facet specific effects, first, Table 5 shows that in situations describing injustice from the victim’s perspective, victim JS had simple effects on all affective responses but guilt as well as on rumination, the anticipation of future injustice, trivialization, perpetrator punishment, victim compensation, social withdrawal, and forgiveness. When all other predictors were controlled for in order to determine the unique effects of victim JS in victim situations, the pattern of effects hardly changed: Victim JS uniquely predicted most variables and the three other JS facets hardly added to these predictions. Only regarding conflict solution, perpetrator JS, but not victim JS had a significant unique effect in this case (Table 5).

Second, Table 6 conveys mixed results when testing the trait activation principle for observer JS. It had simple regression effects on many responses to injustice in situations that were described from the observer’s perspective (all affective responses, rumination, anticipation of future injustice, suppression, victim compensation, conflict solution). These effects, however, vanished almost completely in the multivariate context. Only one unique effect of observer JS remained significant (on sadness). All other responses to observed injustice were better predicted from discording JS facets, particularly victim JS.

**TABLE 6:** Prediction of responses in situations from the observer’s perspective by the JS facets when including only observer JS/all variables.

	Observer situation × victim JS	Observer situation × observer JS	Observer situation × beneficiary JS	Observer situation × perpetrator JS
*Anger/Indignation*	–/0.231***	0.257***/–		
*Guilt*		0.120*/–		
Sadness		0.355***/0.173*		
(Self-)Pity	–/0.166**	0.193**/–		
Disappointment	–/0.229***	0.231*/–		
Helplessness	–/0.279***	0.139*/–		
*Rumination*	–/0.135*	0.339***/–		
Justification		–/–		–/−0.194*
Victim blaming		–/–	–/0.273**	–/−0.272**
Trivialization		–/–		
Suppression		−0.148*/–	–/−0.213*	
Anticipation of future injustice	–/0.203***	0.196***/–		
*Perpetrator punishment*		–/–		–/−0.201*
*Victim compensation*		0.192***/–		
Conflict solution		0.179***/–		
Forgiveness		–/–		
Social withdrawal		–/–		

Only significant path coefficients displayed; ***p < 0.001, **p < 0.01, *p < 0.05 assumed primary responses in italics.

Third, Table 7 shows the results when testing the trait activation principle for beneficiary JS. It had simple regression effects on most responses to injustice in situations that were described from the beneficiary perspective (all affective responses, rumination, anticipation of future injustice, suppression, victim compensation, conflict solution, social withdrawal). When all predictors were jointly considered in the multivariate context, the only remaining unique effects of beneficiary JS were disappointment and victim-blaming, but it continued to add to the prediction of many other responses to injustice from the beneficiary’s perspective beyond additional effects from the discording JS facets.

**TABLE 7:** Prediction of responses in situations from the beneficiary’s perspective by the JS facets when including only beneficiary JS/all variables.

	Beneficiary situation × victim JS	Beneficiary situation × observer JS	Beneficiary situation × beneficiary JS	Beneficiary situation × perpetrator JS
*Anger/Indignation*			0.338***/–	–/0.194*
*Guilt*			0.320***/–	–/0.248**
Sadness			0.366***/–	
(Self-)Pity		–/0.159*	0.197**/–	–/0.170*
Disappointment			0.340***/0.179*	
Helplessness	–/0.153*		0.225***/–	–/0.227*
*Rumination*			0.434***/–	
Justification			–/–	
Victim blaming			–/0.204*	
Trivialization	–/0.232***		−0.252***/−0.264**	
Suppression	–/0.294***		−0.249***/−0.219*	
Anticipation of future injustice	–/0.149*		–/−0.197*	–/0.184*
*Perpetrator punishment*			–/–	
*Victim compensation*			0.332***/0.205*	
Conflict solution	–/−0.159*		0.266***/0.274**	
Forgiveness			–/–	
Social withdrawal			0.111*/–	

Only significant path coefficients displayed; ***p < 0.001, **p < 0.01, *p < 0.05 assumed primary responses in italics.

Fourth, the strongest discrepancy between simple and multiple regression effects emerged for perpetrator JS. As Table 8 shows, the simple effects of perpetrator JS on responses to injustice showed relations with a broad range of responses (all affective responses but anger; helplessness, rumination, justification, victim-blaming, trivialization, suppression, victim compensation, conflict solution). The multivariate analyses did not replicate this pattern. None of the responses to committed injustice was uniquely predicted by perpetrator JS, whereas several effects were detected for the victim (guilt, rumination, forgiveness) and for observer and beneficiary JS (both victim compensation).

**TABLE 8:** Prediction of responses in situations from the perpetrator’s perspective by the JS facets when including only perpetrator JS/all variables.

	Perpetrator situation × victim JS	Perpetrator situation × observer JS	Perpetrator situation × beneficiary JS	Perpetrator situation × perpetrator JS
*Anger/Indignation*				–/–
*Guilt*	–/0.136*			0.263***/–
Sadness				0.214***/–
(Self-)Pity				0.247***/–
Disappointment				0.291***/–
Helplessness				–/–
*Rumination*	–/0.158*			0.300***/–
Justification				−0.154*/–
Victim blaming				−0.147*/–
Trivialization				−0.182**/–
Suppression				−0.118*/–
Anticipation of future injustice				–/–
*Perpetrator punishment*				–/–
*Victim compensation*		–/0.256***	–/−0.203*	0.187**/–
Conflict solution				0.267***/–
Forgiveness	–/0.159*			–/–
Social withdrawal				–/–

Only significant path coefficients displayed; ***p < 0.001, **p < 0.01, *p < 0.05 assumed primary responses in italics.

## Discussion

The present study pursued three main goals. First, we wanted to investigate comprehensively and systematically, which responses unjust situations promote in general and whether potential reactions to injustice depend on the role a person plays in an unjust incident, that is, whether the person experiences the situation as the victim, observer, beneficiary, or perpetrator. Previous studies already found perspective-specific effects but did not simultaneously consider all perspectives and a comparably comprehensive set of potential affective, cognitive, and behavioral reactions. Our study filled this gap by estimating the situation (perspective) main effects for all perspectives and 17 potential reactions to injustice. Our second goal was to more comprehensively investigate the (personality) main effects of the four JS facets on responses to injustice including those that were missing in previous research. Third, we applied trait activation theory to justice and tested the main hypothesis of this theory which predicts, when applied to the justice domain, that JS facets will have stronger effects on affective, cognitive, and behavioral outcomes in unjust situations from the perspective that matches this JS facet. These three research goals are important in themselves, but they also allow a better understanding of the psychological differences between the JS facets and the mechanisms that explain their associations with psychological phenomena, such as internalizing and externalizing problems.

### Main Effects of the Situation

#### General Effects of Injustice

In line with a core assumption of justice theory and previous research (Schmitt, 1996; Mikula et al., 1998), injustice is generally aversive and causes responses that aim to level out injustice. Consequently, the unjust experiences described in the 12 vignettes in the present study elicited a broad range of (partly adverse) affective, cognitive, and behavioral responses, regardless of the perspective from which injustice was perceived and regardless of the individual JS level. Anger, pity, and disappointment were the strongest affective responses across perspectives, rumination and anticipation of future injustice were the strongest cognitive responses, and conflict resolution, victim compensation, and forgiveness were the strongest behavioral responses. These findings are important for future JS research because several of these strong reactions to injustice were not considered in previous studies. They also point to the probability that there are further responses that may be relevant and should be examined. Finally, these findings indicate that the already negative effects of unjust experiences are further exaggerated in individuals high in JS, so that this trait likely places an additional constant burden and strain on these individuals.

#### Perspective Effects

As expected, the unjust scenarios tended to differentially elicit some of the affective, cognitive, and behavioral responses in the present study, depending on the perspective from which these scenarios were described. Other responses, however, were similar in strength across perspectives. For example, affective responses, particularly scenarios from the victim and observer perspective elicited strong responses of anger or indignation; almost no guilt was reported in victim situations, whereas it was a strong affective response in perpetrator situations. In contrast, the average level of helplessness was similar across perspectives. All perspectives were characterized by specific combinations of relevant adverse affective responses, indicating that experiences of injustice require coping irrespective of the perspective from which they are perceived, but may require different coping strategies and result in differential behavioral tendencies. Of note, scenarios from the beneficiary’s perspective were associated with the least pronounced affective responses, indicating that injustice from the beneficiary’s perspective may be less burdensome than that from other perspectives.

Cognitive and behavioral responses showed similar but less pronounced perspective effects than affective responses. Regarding cognitive responses, for example, the average level of reported suppression varied less than that of anticipation of future injustice which was most pronounced in victim situations. Rumination was also similar across perspectives, but also less pronounced for the beneficiary perspective, again indicating that this perspective may be dealt with for example by suppressing too many thoughts about it. Again, all perspectives were characterized by differential patterns of cognitive responses, thereby also indicating different approaches to coping with these situations.

Finally, regarding behavioral responses, forgiveness, and social withdrawal were similar across perspectives, whereas victim compensation more strongly depended on the perspective and was strongest from the perpetrator’s perspective. The pattern of strong behavioral impulses differed between these perspectives as well (Table 3). That is, future research on JS needs to take into consideration the situation from which unjust scenarios are presented, because these perspectives apparently account for parts of the variance.

The pattern of some of the situation effects may not seem surprising to readers familiar with justice research. Nevertheless, it is valuable for at least three reasons. First, its agreement with theoretical expectations and previous research speaks for the construct validity of our scenarios and the utility of our research design. Second, several findings are novel because the responses at issue have not been considered in previous JS research. For instance, sadness, (self-)pity, disappointment, and helplessness are known reactions to injustice but were not included in previous JS research. Similarly, not all cognitive and behavioral responses had been considered in this research but turned out to be relevant in justice-related situations and to create differential patterns depending on the perspective from which injustice was perceived. Thus, future research could take these additional responses into account. Third, some of the present empirical results challenge previous justice theorizing. For instance, for decades, victim-blaming has been claimed to be a preferred strategy to cope with both observed and committed injustice (Sykes and Matza, 1957; Ryan, 1971; Lerner, 1980). Our results do not support this claim. Unexpectedly, victim-blaming was most pronounced in victim situations. Hence, victims are not only harmed by third parties but may even intensify these adverse effects by self-blame. The mean level patterns for social withdrawal also seem important. Beneficiaries and perpetrators who experienced or committed injustice to their advantage and should fear social disapproval reported less social withdrawal than victims and observers who could have been expected to seek social support for their case (observers) or fate (victims) (see below).

### Main Justice Sensitivity (Personality) Effects

Numerous correlations between the JS facets and responses toward injustice in the present study revealed more comprehensive and complex response patterns associated with JS than were previously known. In line with the basic assumptions of the JS construct and previous research, JS was generally related to adverse affective and cognitive responses to unjust situations and to the behavioral responses that were previously assumed, but also to additional ones. Hence, in line with the reasoning outlined above, participants high in JS reported particularly intense responses toward unjust situations, irrespective of the perspective from which injustice was perceived. This finding indicates that individuals high in JS are prone to frequent adverse effects and experiences of strain, irrespective of the facet from which they are sensitive to injustice. This may help to explain the positive associations between all JS facets and internalizing problem behavior as well as strong behavioral responses that aim to level out injustice.

Importantly, all JS facets were positively related to all affective responses in the present study as well as to rumination. In addition, all perspectives but beneficiary JS were related to the anticipation of future injustice. Thus, it can be assumed that these broad adverse affective and cognitive responses and the JS-facet depending patterns of these responses motivate the use of differential mechanisms to cope with unjust experiences and that these differential coping mechanisms account for differential behavioral responses. Of note, victim compensation was consistently associated with all JS facets irrespective of the perspective from which injustice was perceived. This indicates that victim compensation generally is the preferred behavioral response to restore justice, presumably because it may help to cope with the situation at the same time (Table 4).

Importantly, many of these associations are novel findings. Regarding affective responses, this is true for sadness, (self-)pity, disappointment, and helplessness. Our results showed that, on average, they were associated with JS as closely as affective responses that were investigated in previous research (anger, indignation, guilt) and that were considered the primary or most important affective responses. Thus, considering these additional affective responses may help to better understand the complex associations between JS and behavior and well-being. They also show the substantial meaning injustice has for many individuals and indicate the pathways *via* which violations of justice norms may affect behavior and well-being.

Next, cognitive strategies that may otherwise help to overcome adverse emotions associated with injustice, such as trivialization, suppression, or victim-blaming, had previously not been investigated and were shown to be less pronounced among participants who reported higher levels of JS, particularly among those with high perpetrator JS. That is, instead of promoting the tendency to use cognitive strategies of moral disengagement to cope with injustice, high expressions of JS seem to hamper these strategies (see below for a more detailed discussion of this finding).

Finally, the behavioral responses, particularly conflict solution and social withdrawal, had not been and forgiveness had seldom been examined in relation to JS. High altruistic JS was consistently associated with conflict solution, whereas victim JS was also associated with social withdrawal, indicating that it is important to consider these behavioral responses in future research on JS. The positive correlation between victim JS and social withdrawal seems particularly important: In socially withdrawing, victims of injustice may seek to protect themselves from future victimization that is often anticipated by individuals high in victim JS and in situations from the victim’s perspective.

### Person (Justice Sensitivity) × Situation (Perspective) Interaction Effects

Overall, the pattern of associations between the JS facets and the potential responses to injustice was similar regardless of whether all 12 situations were combined (main effects of JS; Table 4) or whether only the situations that matched the perspective of the JS facet at issue were considered (Tables 5–8). This may indicate that the facet from which an individual is sensitive to injustice is even more relevant than the objective perspective from which injustice is perceived. Future research may investigate whether this could be the case because individuals tend to identify with the perspective that is most relevant to them in most situations.

Victim and beneficiary JS, however, showed closer relations with responses in victim and beneficiary situations (simple regression weights in Tables 5, 7), respectively, than in unjust situations in general (Table 4), indicating the expected personality-situation interaction and trait activation effects for these two JS perspectives. Similar trait activation effects were not found for the observer and perpetrator JS. Note that the comparability of the general (Table 4) and the perspective-specific (Tables 5–8) associations are limited because the general outcome included 12 situations whereas the perspective-specific outcomes included only three situations. The potentially less reliable measurement of the perspective-specific outcomes may have made it more difficult to find the accordant effects as compared to the general effects of injustice. This limitation, however, cannot explain why the trait-activation effect remained visible for victim and beneficiary JS, but almost completely disappeared for observer and perpetrator JS as soon as all control variables and discordant JS facets were included in the multiple regression models.

These findings indicate that victim and beneficiary JS may be the most relevant and influential JS facets. Regarding victim JS, this notion is corroborated by the finding that it showed strong and persistent correlations with the different responses in the present study even when considering numerous control variables that also could have explained the effects of victim JS (i.e., trait anger, spite, narcissism, vengeance, moral disengagement). These persistent effects indicate that victim JS is an important trait that may influence behavior and mental health beyond other related variables.

In the present study, out of the three altruistic JS perspectives, beneficiary JS emerged as the most consistent and relevant. It has been argued that this may be the case because being beneficiary sensitive requires individuals to relinquish already gained own benefits although they do not even carry the responsibility for gaining them. That is, beneficiary JS may be more indicative of a genuine concern for justice for the sake of others than even perpetrator JS that “just” requires to avoid injustice in the first place (Bondü and Inerle, 2020). Note, however, that additional effects of victim JS in beneficiary situations suggest that individuals high in beneficiary JS also tend to identify with the victim, further supporting the notion of the central meaning of victim JS.

In observer situations, most responses were better predicted by victim JS than by observer JS (Table 6), suggesting a strong identification of observers with victims of unjust situations (Bondü and Krahé, 2015). This finding may also explain the positive relations between observer JS and reactive aggression in previous research (Bondü and Krahé, 2015; Bondü and Richter, 2016). Identification with another person who is involved in an unjust incident is one mechanism that may explain unexpected effects when all JS facets were simultaneously included in the multivariate prediction models. Mentally adding information from other perspectives to a situation that is described from only one perspective may be another mechanism that may explain the present findings and that is not in accord with the trait-activation principle. For example, passive beneficiaries of injustice may add an active component to the situation if they blame themselves for not having rejected the advantage. This mechanism may explain the unique effects of perpetrator JS in beneficiary situations (Table 7, last column).

In the present study, many of the responses in situations from the perpetrator perspective could not be explained by any of the JS perspectives once all variables were included in the model. First, this may be due to theoretical and empirical overlaps between the three altruistic perspectives that all add to explaining similar parts of variance. Second, in the present study, empathy was a more powerful predictor for some of these responses. This corroborates previous empirical findings that showed positive associations between perpetrator JS and empathy (Edele et al., 2013; Baumert et al., 2014; Strauß et al., 2021) and the notion that the ability to empathize with the potential victim may explain the positive relations between perpetrator JS and prosocial behavior (Strauß et al., 2021). Also note, however, that in recent research, perpetrator JS predicted moral development beyond empathy (Strauß and Bondü, 2022), suggesting that perpetrator JS cannot merely be replaced by empathy but that the two constructs may have differential effects on different outcomes.

### Specific Relations Between Justice Sensitivity Facets and Responses

#### Affective Responses

Affective responses associated with JS include externally directed emotions (anger/indignation, pity) that are more likely to promote behavior to restore justice, and internally directed emotions (sadness, disappointment, helplessness) that are likely to promote social withdrawal (Fetchenhauer and Huang, 2004; Gollwitzer et al., 2015; Bondü, 2018). In line with the theoretical assumptions (Schmitt, 1996), victim JS was most closely related to anger, but also closely associated with disappointment, self-pity, and sadness. Hence, anger may be the first externalizing response to unjust treatment that explains close associations between victim JS and antisocial behavior, particularly when combined with low levels of guilt. There, however, may be a second, internalizing affective response (Downey et al., 1998) that may motivate social withdrawal and explain the relations between victim JS and internalizing problems (Bondü et al., 2017, 2020; Bilgin et al., 2021). Thus, these affective responses should be taken into account by future research when investigating and explaining the relations between victim JS and different outcome measures.

Contrasting previous assumptions, instead of indignation (Schmitt et al., 2005), sadness was the most common affective response among participants high in observer JS, indicating the strong identification with the victim and explaining its consistent correlations with internalizing problems, particularly with eating behavior pathology when combined with feelings of helplessness (Bondü et al., 2020). Similarly, beneficiary JS showed the closest relations with guilt across all unjust situations but was more closely related to anger, sadness, and disappointment in situations from the beneficiary’s perspective. These affective responses are similar to those of the victim, indicating empathy with the victim in highly beneficiary-justice sensitive individuals. This may explain its positive links with perpetrator punishment (Baumert et al., 2014). These findings, therefore, also indicate important differences between beneficiary and perpetrator JS and may help to explain differences between the two facets in behavioral responses. In line with theoretical assumptions (Schmitt et al., 2010), guilt was the strongest affective response toward injustice among participants high in perpetrator JS regardless of the perspective from which injustice was presented, but also pity and disappointment were relevant. Taken together, the broad range of negative affective responses associated with higher levels of JS may explain the links of all JS facets with internalizing problems (Bondü et al., 2017; 2020; Bondü and Inerle, 2020).

#### Cognitive Responses

In line with theoretical assumptions, rumination was the most consistent cognitive response toward injustice (Schmitt et al., 1995) across all JS facets and regardless of the perspective from which injustice was perceived. Rumination is an inadequate cognitive coping strategy (Garnefski et al., 2001) that is frequently associated with depressive symptoms (Hasegawa et al., 2017) and may explain links between JS and internalizing problems (Bondü et al., 2017). Low levels of other cognitive responses that may otherwise help to cope with adverse social experiences, such as justification, victim-blaming, or suppression of thought, among individuals high in altruistic JS also add to explaining their links with internalizing problems.

Taken together, the present findings indicate that high levels of JS increase thoughts about injustice and at the same time hamper the use of cognitive strategies that may help to cope with experiences of injustice, particularly in individuals who would be most in need of these strategies, because they suffer most from the violations of the justice principle. That way, high levels of JS may put extra strain on these individuals. These findings also suggest that particularly individuals high in beneficiary and perpetrator JS may tend to anticipate intense negative affect after unjust situations and, therefore, refrain from antisocial behavior and revert to prosocial behavior (Strauß and Bondü, 2022).

#### Behavioral Responses

Across JS facets and unjust situations, victim compensation was the most common behavioral response (Schmitt et al., 2005, 2010). This may indicate that victim compensation is the mean considered most adequate to restore justice, but also that it best helps to cope with the experience of injustice. It allows to help the victim and counteract injustice, thereby regaining control over the situation. In addition, conflict solution (i.e., pointing out or confessing misbehavior) was a highly relevant behavioral response. Hence, particularly individuals high in observer, beneficiary, and perpetrator JS do not primarily seek perpetrator punishment but tend to give the perpetrator the opportunity to make up for misbehavior. Thus, future research should further investigate this additional behavioral response with regard to JS, for example by shedding light on the ways in which perpetrators can make up for their unjust behavior and which are the prerequisites for forgiveness (e.g., regret).

In contrast, victim JS showed positive associations with perpetrator punishment and negative associations with forgiveness (in situations from the victim’s perspective), supporting previous research that showed positive links between victim JS and antisocial behavior (Schmitt et al., 1995; Fetchenhauer and Huang, 2004; Gollwitzer et al., 2005; Bondü and Krahé, 2015) and negative links with forgiveness (Gerlach et al., 2012). Consequently, it may be particularly important to understand what highly victim-sensitive participants need in order to be able to forgive injustice. Participants high in victim JS, however, were also prone to social withdrawal, suggesting that they may refrain from social interactions to avoid future victimization. Hence, uncooperative and even proactive aggressive behavior by individuals high in victim JS (Gollwitzer et al., 2005, 2009) may be a self-protective strategy in the face of a learned generalized anticipation of further injustice (Fetchenhauer and Huang, 2004; Bondü, 2018), rather than an expression of aggression or a general unwillingness for cooperation.

### Differences Between Justice Sensitivity Facets

Despite some similarities in responses toward unjust experiences across the four JS facets (particularly regarding the affective responses and rumination), our study adds to a better distinction between these facets by carving out numerous facet-specific correlation patterns and correlation sizes. Not surprisingly, differences are most obvious for victim JS in contrast to the three altruistic JS facets. Victim JS was unique by showing the only positive associations with perpetrator punishment and social withdrawal across all unjust situations and negative associations with forgiveness in situations from the victim’s perspective, reflecting a stronger tendency toward adverse behavioral responses. Observer JS differed from the other two altruistic perspectives mainly by higher levels of sadness and the strongest association with victim compensation, again indicating strong identifications with victims.

Importantly, perpetrator JS differed from beneficiary JS by closer relations with guilt, negative associations with perpetrator/self-punishment, and less victim-blaming. The present study indicates that beneficiary JS is a complex JS facet that combines positive effects on behavior with comparably weak affective and cognitive responses and at times even the tendency to explain one’s own advantages (e.g., a positive effect with victim-blaming in situations from the beneficiary perspective when all variables were included; Table 7). Thus, future research may be particularly interested in the contradicting impulses that may signify beneficiary JS.

### Limitations and Outlook

The strengths of the present study include considering a large number of potential responses, a large number of control variables, and the main effects of the unjust situation, the JS facets, as well as their interaction. However, due to time restrictions, we limited the number of potential responses to those that seemed most likely and relevant for all JS facets. There, however, may be further relevant responses (e.g., shame, schadenfreude, mediating between conflicting parties). Further control variables could have been considered. For example, neuroticism may also explain responses associated with JS (e.g., sadness, withdrawal). We used a vignette approach in order to measure potential responses. The reported responses to these scenarios, however, may not fully correspond with those in real-life situations. In addition, we did not use the same three unjust scenarios from all four perspectives, but 12 completely different scenarios. This limits the comparability of perspectives. We used a convenience sample with a large proportion of female and young participants and without *a priori* sample size calculation in our study because many parameters were unknown. Due to a high number of control variables, the present sample may have been too small to uncover all relevant effects. In addition, not all control variables may have been adequately measured, such as extraversion that was only captured by three items. Finally, the present data are only cross-sectional and do not allow for causal inferences.

Future research should, therefore, replicate the present findings in a larger, gender- and age-balanced sample and may include additional measures to assess responses to real-world unjust experiences. We pre-selected scenarios that raters assessed as highly unjust and that should, therefore, be perceived as unjust by both participants high and low in JS. This proceeding allowed us to conservatively test our hypotheses. In contrast, previous research on JS often used ambiguous cues that particularly participants high in JS should interpret as unjust and that would pronounce differences between participants high and low in JS. Finally, one scenario described unintentional unjust treatment (Supplementary material), suggesting that intentionality is no necessary condition for the perception of injustice (Bondü, 2018). Thus, future research may examine the effects of intentionality on responses toward injustice in more detail.

The present study showed that unjust situations trigger a broad range of adverse responses and that, therefore, injustice should be prevented as far as possible. It also showed that individuals high in JS, particularly victim JS, are especially prone to these responses, irrespective of the perspective from which injustice is perceived. Participants high in JS are apparently less able to cognitively diminish the strain associated with unjust experiences. Victim compensation and conflict resolution were the most important behavioral responses; perpetrator punishment was also relevant among participants high in victim JS. Thus, JS can be expected to be a vulnerability and a stress factor at the same time (Bondü et al., 2017). This is particularly true with regard to victim JS in situations that are perceived from the victim’s perspective. These findings help to explain the relations between JS and internalizing and externalizing problems. Thus, JS should be considered a potentially important trait by research on stress, mental health, and social behavior and for prevention and intervention efforts.

## Data Availability Statement

The datasets presented in this study can be found in online repositories. The names of the repository/repositories and accession number(s) can be found below: https://osf.io/ht97r/?view_only=f92d0886340544da83bffeb205f3cd2e.

## Ethics Statement

Ethical review and approval was not required for the study on human participants in accordance with the local legislation and institutional requirements at the time the study was conducted. Written informed consent for participation was not required for this study in accordance with the national legislation and the institutional requirements.

## Author Contributions

RB and DT planned the study design. DT collected the data. RB and AH analyzed the data and drafted the first version of the manuscript. RB and MS revised the manuscript. All authors contributed to the article and approved the submitted version.

## Conflict of Interest

The authors declare that the research was conducted in the absence of any commercial or financial relationships that could be construed as a potential conflict of interest.

## Publisher’s Note

All claims expressed in this article are solely those of the authors and do not necessarily represent those of their affiliated organizations, or those of the publisher, the editors and the reviewers. Any product that may be evaluated in this article, or claim that may be made by its manufacturer, is not guaranteed or endorsed by the publisher.

## Appendix A

### Vignette Examples for Each Justice-Sensitivity Perspective

**Victim:** Your colleague repeatedly tries to give you tasks, although she is not authorized to do so. You tell her that you have enough to do and that you are not willing to take over her tasks. After this your colleague complains about you to your boss. He criticizes your ability to work in a team without listening to your reasons.

**Observer:** You hear about the case of a young neighbor: He took part in a demonstration when an elder resident crossed the place with her shopping bags. With no apparent reason, a police officer threw the woman over and injured her badly. Your neighbor and other demonstrators encircled the police officer and handed him over to his colleagues. Four weeks later your neighbor was charged with opposition to the police. The police officer was not even investigated.

**Beneficiary:** When you exhaustedly arrive in the exhibition center one evening, you search for a hotel room. You have missed a timely reservation. In one hotel you witness a young woman with dreadlocks and sandalsbeing told that all rooms are rented. As you ask for a room again, the receptionist looks at your business attire and says: “For you, of course there is a free room.”

**Perpetrator:** You are a manager and dismissed Mr. Smith without previous notice. He was a loyal and competent employee but got into debt. When 2,000 Euro were missing from the till, you asked all employees to empty their pockets. Mr. Smith had 2,000 Euro with him. He reassured you that he had gotten the money from a friend and even showed a borrower’s note. Nonetheless you did not believe him. Now the accountant points out a calculation error that explains the absence of the 2,000 Euro. Mr. Smith was innocent. Responses towards Injustice Shaped by Justice Sensitivity 35.

## Appendix B

### Potential Affective, Cognitive, and Behavioral Responses Towards Unjust Experiences

**Affect:**

Anger/Indignation: “I would be angry/indignated”
Guilt: “I would feel guilty”
Sadness: “I would be sad”
Disappointment: “I would be disappointed”
(Self-)Pity: “I would feel sorry for the victim/myself”
Helplessness: “I would not know what to do”

**Cognition:**

Rumination: “I would think about the situation for a long time”
Justification: “I would think that the perpetrator/I had good reasons to act like that”
Victim blaming: “I would think that it is the victim’s/my own fault”
Trivialization: “I would tell myself that it is not that bad”
Suppression: “I would try not to think of the situation”
Anticipation: “I would worry that something like that might happen to me one future Injustice day/again”

**Behavior:**

Perpetrator punishment: “I would somehow punish the perpetrator/myself”
Victim compensation: “I would somehow compensate the victim/myself”
Conflict solution: “I would point out the misdemeanor to the perpetrator/I would confess my misdemanor”
Forgiveness: “I would forgive the perpetrator/myself”
Social withdrawal: “I would not trust anyone anymore.”
