# Supplementary material for: Responses Toward Injustice Shaped by Justice Sensitivity – Evidence From Germany
Source: Front Psychol. 2022 Aug 10;13:858291. doi: 10.3389/fpsyg.2022.858291 (PMC9399749; doi:10.3389/fpsyg.2022.858291)
Supplement: Supplementary file 1 [file Data_Sheet_1.PDF]

Supplemantary Material - All Significant Path Coefficients in Models Including all Variables

a) Significant Predictions of Responses in Situations from the Victim's Perspective by all Variables

	Victim JS	Obser-ver JS	Bene-ficiary JS	Perpe-trator JS	Trait Anger	Narcis-sism	Moral Disen-gage-ment	Ven-geance	Spite	Extern. Locus of Control	Intern. Locus of Control	Self-Esteem	Em-pathy	Extra-version	Sex	Ag[illegible]
Anger/Indignation	.324***		-.226**	.194*	.185***					.105*					-.168**	
Guilt			.199**													
Sadness	.175**			.238*											-.203***	
(Self-)Pity	.262***					.125*				.130*			.162**		-.269***	
Disappointment	.282***				.117*					.144**					-.167**	
Helplessness	.156*											-.177*			-.149**	
Rumination	.182**															
Justification	-.189**					.166*	.136*								.136*	
Victim Blaming									.169*			-.186*				
Trivialization	-.231***						.116*	-.132*			-.152*					
Suppression																
Anticipation of Fut. Injustice	.260***								.183**	.197***				-.147**		
Perpetrator Punishment	.213***							.455***		.152**						
Victim Compensation						.224***	.157**	.235***								
Conflict Solution				.218*	.191**	.143*										
Forgiveness	-.238***	.187**				.149*		-.279***	-.131*							
Social Withdrawal	.127*							.138*	.179**			-.156*		-.125*		

Note: Only significant path coefficients displayed; assumed primary responses in italics

b) *Significant Predictions of Responses in Situations from the Observer's Perspective by all Variables*

	Victim JS	Obser-ver JS	Bene-ficiary JS	Perpe-trator JS	Trait Anger	Narcis-sism	Moral Disen-gage-ment	Ven-geance	Spite	Extern. Locus of Control	Internal Locus of Control	Self-Esteem	Em-pathy	Extra-version	Sex	Age
Anger/Indignation	.231***							-.131*		.124*					-.186**	
Guilt										.146*						
Sadness		.173*				.140*							.175**			
(Self-)Pity	.166**												.211***		-.179**	-.177*
Disappointment	.229***							-.140*		.144*						
Helplessness	.279***															
Rumination	.135*						-.162**						.166**			
Justification				-.194*		.174**								-.125*		
Victim Blaming			.273**	-.272**												
Trivialization							.128*			-.170**	.179**					
Suppression			-.213*													
Anticipation of Fut. Injustice	.203***								.143*	.133*						.128*
Perpetrator Punishment				-.201*		.135*		.333***		.181***			.208***			
Victim Compensation					.166*	.228***		.171**								
Conflict Solution						.191**		.147*								
Forgiveness								-.186**								
Social Withdrawal	.								.140*				.215**	-.136*		

Note: Only significant path coefficients displayed; assumed primary responses in italics

c) Significant Predictions of Responses in Situations from the Beneficiary's Perspective by all Variables

	Victim JS	Obser-ver JS	Bene-ficiary JS	Perpe-trator JS	Trait Anger	Narcis-sism	Moral Disen-gage-ment	Ven-geance	Spite	Extern. Locus of Control	Internal Locus of Control	Self-Esteem	Em-pathy	Extra-version	Sex	Age
Anger/Indignation				.194*		.160*									-.140*	
Guilt				.248**						.152*					-.146*	
Sadness					.194**								.198**	-.141*		
(Self-)Pity		.159*		.170*	.171**					.162**			.246***			-.147*
Disappointment			.179*		.211***								.159**			
Helplessness	.153*			.227*				-.141*			-.171*				-.145*	
Rumination						.122*	-.132*			.128*		-.138*				
Justification						.170*	.188**					-.141*		-.150*		.172*
Victim Blaming			.204*			.184**	.120*									
Trivialization	.232***		-.264**				.113*				-.196**					
Suppression	.294***		-.219*													
Anticipation of Fut. Injustice	.149*		-.197*	.184*						.193***	.168**	-.191**		-.117*		
Perpetrator Punishment								.255***		.166**						
Victim Compensation			.205*		.171*				-.163*							
Conflict Solution	-.159*		.274**		.124*	.144*							.176**			
Forgiveness																
Social Withdrawal	.						.117*							-.128*		.145*

Note: Only significant path coefficients displayed; assumed primary responses in italics

d) *Significant Predictions of Responses in Situations from the Perpetrator's Perspective by all Variables*

	Victim JS	Obser-ver JS	Bene-ficiary JS	Perpe-trator JS	Trait Anger	Narcis-sism	Moral Disen-gage-ment	Ven-geance	Spite	Extern. Locus of Control	Internal Locus of Control	Self-Esteem	Em-pathy	Extra-version	Sex	Age
Anger/Indignation					.207**					-.127*						
Guilt	136*												.190**			-.205**
Sadness								-.144*					.175**	-.166**		
(Self-)Pity													.263***			
Disappointment										.103*			.150*			-.124*
Helplessness															-.191***	-.115*
Rumination	.158*											-.147*	154*			
Justification						.235***	.121*									
Victim Blaming					-.249***		.142*	.164*	.160*	.142*						
Trivialization									.156*			.176*				
Suppression									.193**							
Anticipation of Fut. Injustice					.136*					.135*	.159*					
Perpetrator Punishment												-.343***				
Victim Compensation		.256***	-.203*		.184**								.207***			
Conflict Solution													.220***			
Forgiveness	.159*										.173**	.203**				
Social Withdrawal														-.227***		

Note: Only significant path coefficients displayed; assumed primary responses in italics
